# Impact of mobile phone delivered reminders and unconditional incentives on measles-containing vaccine timeliness and coverage: a randomised controlled trial in western Kenya

**DOI:** 10.1136/bmjgh-2020-003357

**Published:** 2021-01-28

**Authors:** E Wangeci Kagucia, Benard Ochieng, Joyce Were, Kyla Hayford, David Obor, Katherine L O'Brien, Dustin G Gibson

**Affiliations:** 1 International Vaccine Access Center, Department of International Health, Johns Hopkins University Bloomberg School of Public Health, Baltimore, Maryland, USA; 2 Kenya Medical Research Institute/Centers for Disease Control and Prevention Public Health and Research Collaboration, Kisumu, Kenya

**Keywords:** measles, vaccines, immunisation

## Abstract

**Introduction:**

Short message service (SMS) reminders coupled with a small monetary incentive conditioned on prompt vaccination have been shown to improve first-dose measles-containing vaccine (MCV1) uptake. We assessed whether SMS reminders and unconditional monetary incentives—more amenable to programmatic implementation—can improve MCV1 uptake in Kenya.

**Methods:**

Caregivers of eligible infants aged 6–8 months were enrolled into an individually randomised controlled trial and assigned to receive either: no intervention (control), two SMS reminders (SMS) sent 3 days, and 1 day before the scheduled MCV1 date, or SMS reminders coupled with a Kenya Shilling (KES) 150 incentive (SMS +150 KES) sent 3 days before the scheduled MCV1 date. Study staff conducted a household follow-up visit at age 12 months to ascertain vaccination status. Log-binomial regression was used to estimate the relative and absolute difference in MCV1 timely coverage (by age 10 months), the primary outcome.

**Results:**

Between 6 December 2016 and 31 March 2017, 179 infants were enrolled into each of the three study arms. Follow-up visits were completed between 19 April 2017 and 8 October 2017 for control (n=170), SMS (n=157) and SMS + 150 KES (n=158) children. MCV1 timely coverage was 68% among control arm infants compared with 78% in each intervention arm. This represented a non-statistically significant increase in the SMS arm (adjusted relative risk 1.13; 95% CI 0.99 to 1.30; p=0.070; adjusted risk difference 9.2%; 95% CI: −0.6 to 19.0%; p=0.066), but a statistically significant increase in the SMS + 150 KES arm (1.16; 95% CI 1.01 to 1.32; p=0.035; 10.6%; 95% CI 0.8 to 20.3%; p=0.034).

**Conclusion:**

These findings suggest that the effect of SMS reminders coupled with a small unconditional monetary incentive on MCV1 uptake is comparable to that of SMS reminders alone, limiting their utility. Further studies in the absence of unexpected supply-side constraints are needed.

**Trial registration number:**

NCT02904642

**Summary box**

**What is already known?**

- In low-income and middle-income country (LMIC) settings, short message service (SMS; text message) reminders alone can significantly improve timeliness of first dose measles-containing vaccine (MCV1) receipt—that is, vaccination within a short window of the recommended age—but evidence of their impact on the overall proportion of infants receiving MCV1 is mixed.
- The effect of SMS reminders coupled with a small conditional monetary incentive on MCV1 timeliness and MCV1 coverage by age 12 months appears to be superior to that of SMS reminders alone.
- Unconditional incentives may be more feasibly delivered compared with conditional incentives but their effectiveness under real-world conditions within LMIC immunisation programmes is unknown.

**What are the new findings?**

- SMS reminders coupled with a small unconditional monetary incentive improved MCV1 timeliness but not MCV1 coverage by age 12 months in rural Kenya.
- The magnitude of the effect of SMS reminders coupled with an unconditional incentive on MCV1 timeliness and MCV1 coverage by age 12 months was similar to that of SMS reminders alone.

**What do the new findings imply?**

- These findings suggest no added effect of small unconditional monetary incentives over that of SMS reminders alone. However, the findings may have been limited by supply-side barriers to vaccination and additional studies are needed to validate the findings.
- This study adds to the body of evidence on the combined effect of SMS reminders and incentives; it is the second to evaluate the effect SMS reminders coupled with any kind of monetary incentive and the first to assess the effect of SMS reminders coupled with unconditional incentives.

## Introduction

Measles vaccination is estimated to have prevented 1.3 million global deaths annually between 2000 and 2018 and to have decreased annual measles incidence by approximately two-thirds in the same period.^1^ However, the full potential of measles-containing vaccine (MCV) to prevent morbidity and mortality remains unrealised. Failure to achieve measles control targets, including elimination, is largely attributable to suboptimal measles vaccination uptake.

In 2019, global coverage for the first and second doses of MCV was 85% and 71%, respectively,^2^ falling below the threshold of 95% coverage with two MCV doses needed for measles elimination.^3^ Inequalities in vaccination coverage at the global level—for example, first-dose MCV (MCV1) coverage in 2019 was 69% in WHO Africa Region^4^ compared with >90% in the WHO Europe, South East Asia and Western Pacific Regions^5–7^—and at the subnational level are widely acknowledged.^8 9^

Given high levels of mobile phone ownership and access in low-income and middle-income countres (LMICs)—in 2017 there were 99 and 77 mobile phone subscribers per 100 inhabitants in developing and African countries, respectively^10^—mobile phone-based interventions may improve demand for routine MCV. Indeed, short message service (SMS; text message) reminders alone or coupled with other interventions have been shown to: increase uptake of diphtheria, tetanus and pertussis (DTP) containing vaccine^11–19^; increase uptake of MCV^17 19 20^; improve full immunisation coverage (FIC)^12 17–19^; and improve vaccine-seeking in general^21^ among caregivers of infants in a variety of LMICs. In addition, SMS reminders coupled with mobile phone-based incentives (airtime and mobile money (mMoney)) have been shown to improve uptake of DTP-containing vaccine, uptake of MCV, FIC^17^ and age-appropriate vaccination coverage^22^ among children in LMICs.

Previously, we showed that SMS reminders coupled with small, conditional mMoney incentives significantly improved measles vaccination timeliness (ie, the proportion of children vaccinated within 2 weeks of the vaccine due date) and measles vaccination coverage (ie, the proportion of children receiving measles vaccination by age 12 months) in a cluster randomised controlled trial conducted in Kenya. In that study, incentives were conditioned on receipt of measles vaccination within 2 weeks of the recommended age.^17^ Scaling up SMS reminders coupled with conditional incentives in Kenya may not be feasible as it would require real-time monitoring of vaccination receipt in order to determine if caregivers meet the conditions for receiving the incentive. In turn, real-time monitoring of vaccination receipt under real-world conditions is challenged by limitations in the human and/or financial resources needed to implement it. Thus, unconditional incentives which do not require monitoring of vaccination receipt prior to delivery, may be more feasibly delivered at scale to improve measles vaccination coverage.

We sought to evaluate the impact of SMS reminders coupled with an unconditional mMoney incentive on MCV1 timeliness and coverage in Kenya.

## Methods

### Study design and participants

The Mobile and Scalable Innovations for Measles Immunisation (M-SIMI) study was a three-arm parallel individually randomised controlled trial conducted in Gem subcounty, Siaya County, Kenya. Gem subcounty is a predominantly rural setting with a population of approximately 164 000 in 2016^23 24^ characterised by relatively high malaria, HIV and tuberculosis prevalence and high infant mortality.^25^ Vaccination coverage was over 90% for third dose DTP-containing vaccine (DTP3) and 84% for MCV1 by age 12 months in 2014–2015.^17^ The study was conducted in areas within the Kenya Medical Research Institute and Centers for Disease Control and Prevention collaboration’s Health and Demographic Surveillance System (HDSS).

Eligible infants were required to: be aged 6–8 months; be residents of the subcounty as reported by the caregiver and to not have received a dose of routine measles vaccine as indicated in the home-based vaccination record (maternal and child health booklet). Infants’ caregivers were required to not have plans to move within 6 months of enrolment. Mobile phone ownership by the caregiver was not a requirement for enrolment. Participants were randomised and evenly allocated to one of three study arms: (1) control, (2) SMS reminders (SMS), (3) SMS reminders plus a 150 Kenya Shillings incentive (KES; SMS+150; KES150=US$1.50 as of December 2016).

The conduct, analysis and reporting of results were conducted in accordance with the Consolidated Standards of Reporting Trials guidelines.^26^A detailed description of the methods and protocol has been reported.^27^

### Randomisation and masking

Simple randomisation with an allocation ratio of 1:1:1 to the control, SMS or SMS +150 arm was performed using a list of computer-generated random numbers. Randomisation and preparation of the allocation envelopes were done by the data manager who had no contact with participants. Given the nature of the interventions, study field staff and participants were not blinded to the study arm. The data analyst had access to participants’ study arm allocation during analysis. Additional details on the allocation procedure are provided in the supplement.

### Procedures

Community health volunteers (CHVs)—a component of Kenya’s national Community Health Strategy—identified households with children aged 6–8 months and relayed this information to study-employed Community Interviewers (COMM-Is). COMM-Is then visited households to provide general information about the study and to perform screening procedures. COMM-Is verified age eligibility using the date of birth recorded in the home-based vaccination record. Written informed consent was obtained for eligible caregivers. Immediately after enrolment, COMM-Is collected vaccination status, sociodemographic, economic, mobile phone access, mobile phone usage, healthcare utilisation and other general health information from caregivers. Caregivers who did not own a phone were asked to confirm a phone number to which SMS reminders and the mMoney incentive (as applicable) could be sent. Caregivers who could not identify a shared phone number for the study were offered the option to use the COMM-I’s work phone number. All participants received an enrolment SMS, which included a health-related motivational phrase.^27^

Control arm participants received no interventions. SMS arm participants were sent two SMS reminders; one 3 days before the scheduled MCV1 date (3-day reminder) and the other 1 day before the scheduled measles vaccination date (1-day reminder). SMS+ 150 KES arm participants were sent reminders on the same schedule as the SMS arm participants and were sent the KES 150 incentive 3 days before the scheduled measles vaccination date that is, on the same day as the 3-day reminder.

SMS reminders were sent in the caregiver’s preferred language that is, Dholuo, Kiswahili or English, as indicated at enrolment. The 3-day reminder was comprised of a standard reminder portion, a phrase intended to motivate caregivers and, for SMS+150 KES arm participants, language explaining that the study was sending the incentive to assist with travel expenses. The 1-day reminder was the same across intervention arm participants and consisted of a reminder portion as well as a motivational phrase that was different from the 3-day reminder motivational phrase (online supplemental box S1).^27^ Caregivers in the control arm were expected to receive one text message (enrolment message only) total and those in the intervention arms were expected to receive three text messages total.

10.1136/bmjgh-2020-003357.supp1 Supplementary data

SMS reminders were sent out automatically using RapidSMS, an open-source platform.^28^ At enrolment, COMM-Is submitted an SMS to the RapidSMS server containing the infant’s name, infant’s date of birth, and caregiver’s preferred phone number. Based on the information submitted, the 3-day and 1-day SMS reminders, tailored to the applicable study arm and including the infants’ name, were sent from the RapidSMS server to the phone number provided by the caregiver. The Kenya Expanded Programme on Immunisation recommends MCV1 administration at age 9 months.^29^ Thus, the scheduled measles vaccination date was 274 days (30.42 days/month) from the infant’s date of birth, if falling on a weekday. If falling on a Saturday or Sunday, the scheduled measles vaccination date was defined as the following Monday. RapidSMS was programmed to send reminder messages to a study phone to allow monitoring of per-protocol transmission of SMS. Logs of sent SMS were generated from RapidSMS.

RapidSMS was also programmed to automatically create a cumulative incentive payment list for infants enrolled in the SMS + 150 KES arm. The payment list included infants’ Study IDs, caregivers’ preferred phone numbers and payment dates (ie, 3 days before the scheduled vaccination date). Using the RapidSMS-generated payment list, study staff manually transmitted the KES 150 incentive from a smart phone using the M-PESA mobile money platform operated by Safaricom, one of Kenya’s mobile network providers.

COMM-Is administered a follow-up survey when infants were aged 12 months to collect vaccination status as well as information on caregivers’ opinion of the interventions, reasons for delayed measles vaccination (ie, not vaccinated by age 10 months), incentive use and other general health information. If the child’s vaccinations were not up to date at the follow-up visit, the COMM-I referred the caregiver to the nearest health facility for vaccinations.

### Outcomes

The primary outcome was the proportion of infants receiving MCV1 by age 10 months (304 days; ie, MCV1 timely coverage). Secondary outcomes were the proportion of infants receiving MCV1 by age 12 months (365 days; that is, MCV1 coverage) and time to measles vaccination by age 12 months. At the follow-up visit, vaccination status was ascertained from either the home-based vaccination record or the caregiver’s verbal report if a home-based vaccination record was not available. If the home-based vaccination record was available, the date (day, month and year) of vaccination was transcribed and used to calculate the infant’s age at vaccination. For verbal vaccination reports, caregivers specified the month and year of vaccination.

### Statistical analysis

The study aimed to measure a≥15 percentage point absolute increase in MCV1 timely coverage in the intervention arms compared with the control arm. We presumed that a≥15% increase in the proportion of children receiving MCV1 by age 10 months would represent a meaningful effect from a policy-maker perspective. Based on previous coverage estimates in the study area,^17^ we assumed MCV1 timely coverage of 70% in the M-SIMI control group. We also assumed a type 1 error (alpha) of 0.05, a power (1-beta) of 0.80, yielding a sample size of 134 infants per study arm after application of a continuity correction. The sample size was adjusted to account for up to 25% lost to follow-up, which included death, outmigration and verbal report of measles vaccination at 10 months of age. A priori, verbal immunisation reports at the follow-up visit were to be excluded from the analytic sample. After accounting for potential losses to follow-up, the estimated sample size to assess the primary outcome was 537 infants total, or 179 infants enrolled per study arm.

Log-binomial regression was used to estimate the relative risk (RR), difference in risk (RD) and respective 95% CI of measles vaccination by age 10 months or age 12 months in each of the intervention arms compared with control arm. For assessment of the primary endpoint (MCV1 timely vaccination), children were censored at age 10 months. Any predictive baseline characteristics determined to be unequally distributed at the 5% significance level were included as covariates in the regression model to adjust for potential confounding. The primary endpoint was analysed according to intention-to-treat (ITT) principles. A modified per-protocol sensitivity analysis for the primary endpoint was also conducted. The modified per-protocol analysis was defined as dispatch of two SMS reminders as per the target schedule that is, 3 days and 1 day before the scheduled vaccination date.

In order to evaluate whether baseline participant characteristics modified the effect of the interventions on the likelihood of timely measles vaccination, we performed stratified (subgroup) analysis. To identify independent variables to include in the subgroup analysis, a risk factor analysis of baseline participant characteristics associated with MCV1 vaccination by age 10 months was conducted among only control arm participants using univariate log-binomial regression. Characteristics significant at the 10% level were included in subgroup analysis. Stratification by mobile phone ownership status and travel time to the health facility were pre-specified in the study protocol. Stratification by maternal education was added post hoc, based on evidence of an association between higher educational attainment and lower likelihood of missed measles vaccination in the study area.^17^ The significance level for subgroup analysis was 5%.

Survival analysis was performed to assess whether time to measles vaccination differed significantly across the study arms. Time origin was defined as enrolment and events were right censored at age 365 days. The cumulative probability of measles vaccination was plotted using the failure functions estimated using the Kaplan-Meier method. Equality of the cumulative incidence functions were tested using the log-rank test.

Analyses were performed using Stata/SE, V.14.1 (StataCorp).

### Patient and public involvement

This study did not involve patients. We did not directly include participants or public representatives in the design of this study, but the design was informed by focus group discussions among community members for a similar, related study previously conducted in the same area.^30^ No participants or public representatives contributed to selection of outcome measures. CHVs, who are community members, were involved in identification of study participants. Study findings were presented to CHVs.

## Results

**Figure 1:**
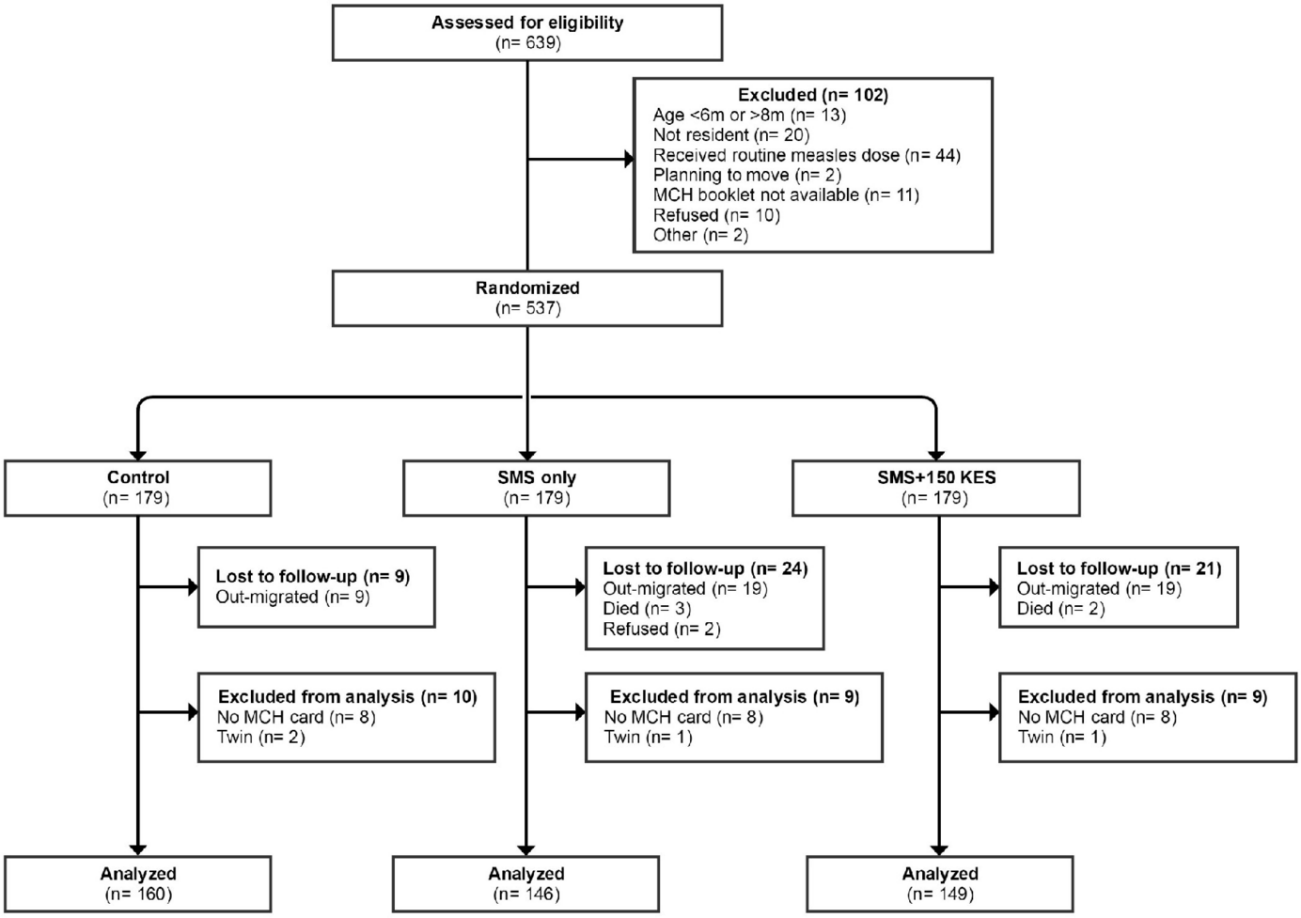
Screening, enrolment and follow-up flow diagram for the M-SIMI study. M-SIMI, Mobile and Scalable Innovations for Measles Immunisation; SMS, short message service; MCH card, maternal and child health card (home-based vaccination record).

CHVs identified 639 potentially eligible infants between 6 December 2016 and 31 March 2017. Of those, 537 infants—the target sample size—were randomised to the control, SMS and SMS + 150 KES arms (179 infants each). Follow-up visits were completed for control (n=170), SMS (n=157) and SMS + 150 KES (n=158) infants between 19 April 2017 and 8 October 2017. The analytical sample included 160 (89%), 146 (82%) and 149 (83%) of all infants enrolled in the Control, SMS and SMS + 150 KES arms, respectively. Reasons for exclusion from enrolment, loss to follow-up and exclusion from the analytical sample are provided in figure 1. The 82 participants who were excluded from the analytical sample were similar to participants in the analytic sample except for birth order and maternal age (online supplemental table S1). Maternal age was included as an independent variable in regression models because it was unevenly distributed across study arms (table 1) and has previously been shown to be a determinant of childhood vaccination status within the study area.^31^ The per-protocol analysis included 126 SMS and 126 SMS + 150 KES infants, representing 86% and 85% of the analytical sample, respectively.

**Table 1:** Baseline characteristics of infants in the analytical sample, Gem subcounty, Kenya (2016–2017)

	Control (n=160) (%)	SMS (n=146) (%)	SMS + 150KES (n=149) (%)	Total (n=455) (%)
Mobile phone access				
Shares	49 (31)	48 (33)	46 (31)	143 (31)
Owns	111 (69)	98 (67)	103 (69)	312 (69)
Infant’s sex				
Female	77 (48)	70 (48)	69 (46)	216 (48)
Male	83 (52)	76 (52)	80 (54)	239 (53)
Infant’s age at enrolment				
6 months	104 (65)	92 (63)	96 (64)	292 (64)
7 months	53 (33)	52 (36)	51 (34)	156 (34)
8 months	3 (2)	2 (1)	2 (1)	7 (2)
Penta3 before enrolment				
Not vaccinated	6 (4)	7 (5)	6 (4)	19 (4)
Vaccinated	154 (96)	139 (95)	143 (96)	436 (96)
Time to health facility				
≤30 min	110 (69)	85 (58)	97 (65)	292 (64)
>30 min	50 (31)	61 (42)	52 (35)	163 (36)
Maternal education				
≤7 years	53 (33)	54 (37)	49 (33)	156 (34)
>7 years	107 (67)	92 (63)	100 (67)	299 (66)
Birth order				
Firstborn	33 (21)	31 (21)	22 (15)	86 (19)
Later born	127 (79)	115 (79)	127 (85)	369 (81)
Location of last delivery				
At home	30 (19)	28 (19)	25 (17)	83 (18)
Health facility	129 (81)	118 (81)	124 (83)	371 (82)
Maternal age				
≤25 years	80 (50)	86 (59)	62 (42)	228 (50)
>25 years	80 (50)	60 (41)	87 (58)	227 (50)
No of ANC visits for enrolled infant				
≤4 visits	114 (71)	99 (68)	96 (65)	309 (68)
>4 visits	46 (29)	47 (32)	51 (35)	144 (32)
Socioeconomic quintile				
Bottom 40%	72 (45)	55 (38)	52 (35)	179 (39)
Upper 60%	88 (55)	91 (62)	97 (65)	276 (61)

Data are n (%).

ANC, antenatal care; KES, Kenya Shillings; Penta3, third-dose pentavalent vaccine; SMS, short message service.

**Figure 2:**
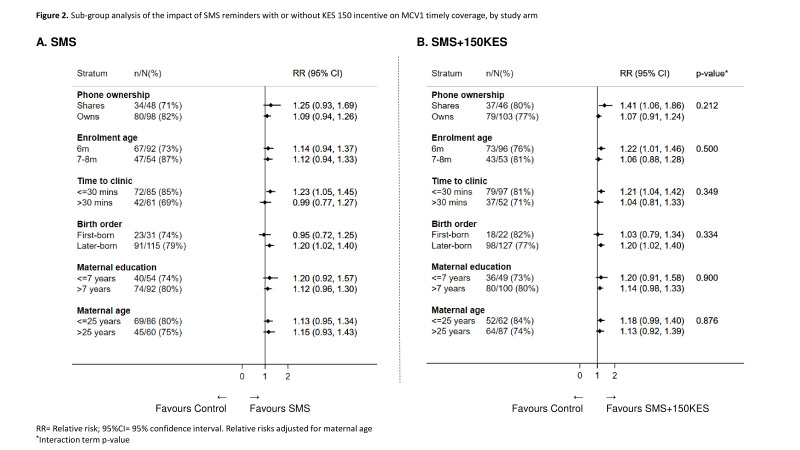
Subgroup analysis of the impact of SMS reminders with or without KES 150 incentiveon MCV1 timely coverage, by study arm. Relative risks (RR) adjusted for maternal age* Interaction term p value. KES, Kenya Shilling; MCV, measles-containing vaccine; SMS, short message service.

MCV1 timely coverage was 68% (109 of 160) among Control arm infants compared with 78% (114 of 146 in SMS only; 116 of 149 in SMS + 150 KES) in both the intervention arms. This represented a non-statistically significant increase in the SMS arm (adjusted RR (aRR) 1.13; 95% CI 0.99 to 1.30; p=0.070; adjusted risk difference (aRD) 9.2%; 95% CI −0.6 to 19.0%; p=0.066), but a statistically significant increase in the SMS + 150 KES arm (aRR 1.16; 95% CI 1.01 to 1.32; p=0.035; aRD 10.6%; 95% CI 0.8 to 20.3%; p=0.034; table 2). In the per-protocol analysis, MCV1 timely coverage in the intervention arms was similar to ITT MCV1 timely coverage and findings were comparable to the ITT analysis with the exception that the absolute increase in MCV1 timely coverage among SMS infants achieved statistical significance (table 2). None of the characteristics identified in the analysis of predictors of vaccination in the Control arm (phone ownership, age at enrolment, birth order and time to health facility; online supplemental table S2) modified the effect of the interventions on MCV1 timely coverage. Post hoc, maternal age was added to the subgroup analysis and similar to the other variables assessed, was not found to modify the effect of the interventions (figure 2). MCV1 coverage was not significantly higher in the SMS (84%; 125 of 160) or SMS + 150 (85%; 123 of 146) arms compared with the Control arm (78%; 126 of 149; table 2).

**Table 2:** Measles vaccination timeliness by age 10 and coverage by age 12 months in intervention arms compared with the control arm

	N	No vaccinated (%)	RR (95% CI)	Adjusted RR (95% CI)*	P value	RD (95% CI)	Adjusted RD (95% CI)*	P value
*MCV1 at 10 months*								
Intention-to-treat analysis								
Control	160	109 (68)	Ref	Ref	–	Ref	Ref	–
SMS	146	114 (78)	1.15 (1.01 to 1.32)	1.13 (0.99 to 1.30)	0.070	10.0 (0.1 to 19.8)	9.2 (−0.6 to 19.0)	0.066
SMS +150KES	149	116 (78)	1.14 (1.00 to 1.31)	1.16 (1.01 to 1.32)	**0.035**	9.7 (−0.1 to 19.5)	10.6 (0.8 to 20.3)	**0.034**
Per-protocol analysis								
Control	160	109 (68)	Ref	Ref	–	Ref	Ref	–
SMS	126	100 (79)	1.16 (1.01 to 1.34)	1.15 (1.00 to 1.32)	0.052	11.2 (1.1 to 21.3)	10.2 (0.1 to 20.3)	**0.048**
SMS +150KES	126	99 (79)	1.15 (1.00 to 1.33)	1.17 (1.02 to 1.35)	**0.024**	10.4 (0.3 to 20.6)	11.6 (1.4 to 21.7)	**0.025**
*MCV1 at 12 months*								
Intention-to-treat analysis								
Control	160	125 (78)	Ref	Ref	–	Ref	Ref	–
SMS	146	123 (84)	1.08 (0.97 to 1.20)	1.07 (0.96 to 1.19)	0.199	6.1 (−2.6 to 14.8)	5.7 (−3.0 to 14.3)	0.199
SMS + 150KES	149	126 (85)	1.08 (0.97 to 1.20)	1.09 (0.97 to 1.20)	0.156	6.4 (−2.2 to 15.1)	6.8 (−1.8 to 15.3)	0.119
Per-protocol analysis								
Control	160	125 (78)	Ref	Ref	–	Ref	Ref	–
SMS	126	107 (85)	1.09 (0.97 to1.21)	1.08 (0.97 to1.20)	0.163	6.8 (−2.2 to 15.7)	6.3 (−2.6 to 15.2)	0.165
SMS + 150KES	126	106 (84)	1.08 (0.96 to1.20)	1.08 (0.97 to1.21)	0.166	6.0 (−0.3 to 15.0)	6.4 (−2.5 to 15.3)	0.161

Bold indicates p<0.05.

*RR and RD adjusted for maternal age.

RR, relative risk; RD, risk difference; MCV1, measles-containing vaccine; SMS, short message service; KES, Kenya Shillings; Ref, reference group.

**Figure 3:**
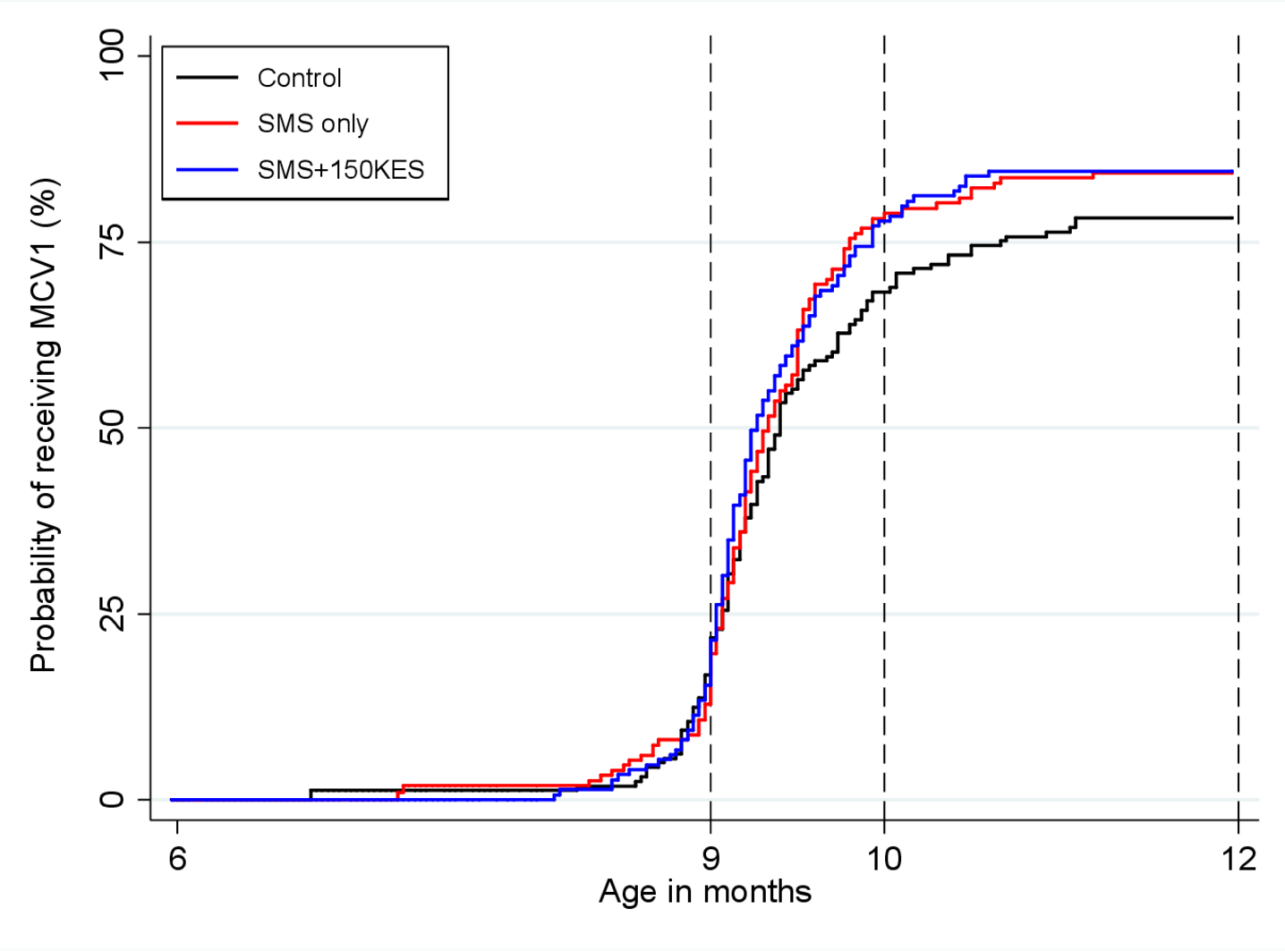
Cumulative incidence of measles vaccination by age 12 with time origin as age at enrolment. MCV, measles-containing vaccine; SMS, short message service.

The median time to measles vaccination was age 286 days (IQR 276–324 days) in the control arm, age 284 days (IQR 276–298 days) in the SMS arm and age 282 days (IQR 275–302 days) in the SMS + 150 KES arm (figure 3). There was no significant difference in time to measles vaccination across study arms (log-rank test p=0.182; online supplemental figure S1) even after performing testing stratified by maternal age (maternal age ≤25 years log-rank test p=0.195, maternal age >25 years log-rank test p=0.576, stratified log-rank test p=0.158; data not shown).

The reason for delayed vaccination was obtained for 82% (95 of 116) of infants with delayed MCV1. Caregivers who were not queried were missed because the interviewer did not identify them as having delayed vaccination. Of the infants whose caregivers were queried, common (>10%) reasons for not receiving MCV1 by age 10 months were an ongoing nurses strike (36%) and vaccine stock-out (16%). Other reported reasons for delayed vaccination are provided in table 3. There was no significant difference in cause of delayed vaccination across study arms (overall χ^2^ p=0.529).

**Table 3:** Reasons for delayed measles vaccination

	Control (N=160) n (%)	SMS (N=146) n (%)	SMS + 150KES (N=149) n (%)	Total (n=455) n (%)
Not vaccinated by age 10 months	51 (32)	32 (22%)	33 (22)	116 (26)
Reason for delay queried	41 (80)	29 (91)	25 (76)	95 (82)

Reason for delay	Control (N=41) (%)	SMS (N=29) (%)	SMS + 150KES (N=25) (%)	Total (N=95) (%)
Nurses' strike	12 (29)	13 (45)	9 (36)	34 (36)
Vaccine not in stock	6 (15)	4 (14)	5 (20)	15 (16)
Child was ill	4 (10)	2 (7)	2 (8)	8 (8)
Reason not given	1 (2)	4 (14)	2 (8)	7 (7)
Travelling	4 (10)	2 (7)	1 (4)	7 (7)
Not recorded in home-based vaccination record	5 (12)	0 (0)	1 (4)	6 (6)
Didn't know date	3 (7)	0 (0)	2 (8)	5 (5)
Nurse refused to open vial	2 (5)	0 (0)	1 (4)	3 (3)
Forgot	1 (2)	1 (3)	0 (0)	2 (2)
Competing priorities	1 (2)	1 (3)	0 (0)	2 (2)
Clinic too far	0 (0)	0 (0)	1 (4)	1 (1)
Previous vaccine delayed	1 (2)	0 (0)	0 (0)	1 (1)
Vaccine not important	0 (0)	0 (0)	1 (4)	1 (1)
Forgot home-based vaccination record	0 (0)	1 (3)	0 (0)	1 (1)
Discouraged by friend	1 (2)	0 (0)	0 (0)	1 (1)
Caretaker was ill	0 (0)	1 (3)	0 (0)	1 (1)

SMS, short message service; KES, Kenya Shillings.

Reasons why reminders were not sent per-protocol are shown in online supplemental table S3. Whereas at least one SMS reminder was sent out by the study team to participants, 67% (98 of 146) and 81% (120 of 149) of SMS arm and SMS + 150 KES arm caregivers, respectively, reported that they received at least one SMS reminder. The proportion of caregivers who did not know the total number of reminders received was significantly higher among those sharing a phone compared with those who owned the phone receiving messages (table 4). Due to an error in the questionnaire skip pattern, caregivers who reported not receiving any reminders were not queried as to whose phone the reminders were sent. More than 85% of caregivers in each of the intervention arms felt that SMS reminders influenced the decision to vaccinate (table 4). mMoney incentives were sent to all participants in the SMS +150 KES arm. Ninety-one (61%) incentives were sent out 3 days before the scheduled vaccination date, as intended in the study protocol, and 127 (85%) before the scheduled vaccination date (online supplemental table S4). Of 149 SMS + 150 KES arm caregivers, 71% (n=105) reported that they received the mMoney incentive, of whom 72% (76 of 105) owned the phone to which the mMoney incentive was sent. Receipt of the incentive was reported to have influenced the decision to vaccinate in 84% (88 of 105) of these caregivers. A little over three-quarters of caregivers cashed out the incentive within 3 days of receiving the incentive. Among infants whose caregivers who cashed out the incentive, 19% (20 of 103) and 13% (13 of 103) did not receive MCV1 by age 10 months and age 12 months, respectively (online supplemental table S5). Approximately 95% of caregivers reported a positive experience related to receiving the incentive. Only one caregiver reported that they would be less likely to seek vaccination in the absence of an incentive. Incentives were commonly used to cover transport costs (n=59; 56%), housing expenses (n=21; 20%) and to purchase food (n=16; 15%; table 4).

**Table 4:** Reported receipt of SMS reminders, opinions about SMS reminders, experience with and opinions about incentives

Reported receipt of SMS reminders								
	SMS (N=146)				SMS + 150 KES (N=149)			
	All n (%)	Own n (%)	Share n (%)	P value	All n (%)	Own n (%)	Share n (%)	All n (%)
Received ≥1 SMS reminder	98 (67)	66 (67)	32 (33)	–*	120 (81)	82 (68%)	38 (32)	–*
Received one reminder	30 (31)	18 (27)	12 (38)	0.303	21 (18)	12 (15%)	9 (24)	0.225
Received two reminders	62 (63)	47 (71)	15 (47)	**0.019**	94 (78)	68 (83%)	26 (68)	0.073
Received three reminders	1 (1)	1 (2)	0 (0)	0.484	2 (2)	2 (2%)	0 (0)	0.332
Don’t know	5 (5)	0 (0)	5 (16%)	**0.001**	3 (3)	0 (0)	3 (8)	**0.010**
Opinions about SMS reminders								
	**SMS** (**N=98**) (%)				**SMS + 150KES** (**N=120**) (%)			
SMS influenced decision to vaccinate	88 (90)				110 (92)			
Received MCV1 by age 12 months	80 (91)				96 (87)			
Number of SMS reminders								
Too few	8 (8)				15 (13)			
Just right	80 (82)				94 (78)			
Shared mobile phone	7 (7)				9 (8)			
Don't know	3 (3)				2 (2)			
Length of SMS reminders								
Too short	0 (0)				1 (1)			
Right length	88 (90)				106 (88)			
Shared mobile phone	8 (8)				13 (11)			
Don't know	2 (2)				0 (0)			
Experience with, and opinions about incentives								
					**SMS +150 KES (N=149**) (%)			
**Received MCV1 incentive**					105 (71)			
Owned phone					76 (72)			
Incentive influenced decision to vaccinate					88 (84)			
Received MCV1 by age 12 months					78 (89)			
Mobile money cashed out								
Day when received					16 (15)			
Within 1–3 days of receipt					67 (64)			
More than 3 days after receipt					20 (19)			
Not cashed out					2 (2)			
Experience receiving incentive								
Very positive					98 (93)			
Somewhat positive					2 (2)			
Neutral					4 (4)			
Very negative					1 (1)			
Likelihood of future vaccination in the absence of incentive								
More likely					95 (91)			
Less likely					1 (1)			
The same					8 (8)			
Don't know					1 (1)			
M-PESA use								
Transport cost					59 (56)			
Housing expenses					21 (20)			
Food					16 (15)			
Airtime					1 (1)			
Medicine					5 (5)			
Infant’s clothing					2 (2)			
Not used yet					1 (1)			

*p value not calculated as ownership of phone number to which reminder was sent was only collected for participants who reported that they received a reminder; there is no corresponding data on phone ownership for participants who reported that they did not receive any reminders.

KES, Kenya Shillings; MCV, measles-containing vaccine; SMS, short message service.

## Discussion

When delivered under real-world conditions, SMS reminders coupled with a small unconditional monetary incentive increased the timeliness of MCV1 receipt, as did SMS reminders despite not achieving statistical significance. However, MCV1 timely coverage was significantly higher in the SMS arm compared with the control arm in the per-protocol analysis, suggesting that when delivered under ideal conditions, SMS reminders alone may improve MCV1 timeliness. Though the effect of the interventions on MCV1 timely coverage was modest—the findings suggested the potential to improve timeliness by 9%–11% points—the magnitude of effect could be meaningful from a policy perspective as delay in vaccination increases the proportion of susceptible persons, of particular clinical concern in high HIV prevalence settings^32^ such as this one. There was no evidence that the interventions exacerbated or reduced inequities in MCV1 timeliness. The effects of the interventions on MCV1 coverage by age 12 months were even more modest and did not achieve statistical significance, leaving unclear whether either intervention may reduce the proportion of infants not receiving routine MCV.

This study was novel in that it evaluated the combined impact of SMS reminders and unconditional monetary incentives on vaccination timeliness and coverage. To our knowledge, it is the first study to evaluate the combined effect of those interventions. It is only the second study, following our previous evaluation that provided conditional monetary incentives,^17^ to evaluate the impact of SMS reminders coupled with any type of monetary incentive on vaccination uptake; other studies have assessed the impact of SMS reminders^11–16 18 20 33 34^ and incentives separately.^35–37^ Furthermore, we illustrated that CHVs can support community-based identification of infants targeted for immunisation interventions.

The finding from this study that SMS reminders coupled with an incentive significantly increase measles vaccination timeliness is consistent with the M-SIMU study^17^ and reproduces a positive finding within the same study population. However, the findings also suggest differential impact of unconditional *vs* conditional incentives on MCV1 uptake. First, the magnitude of the impact of SMS reminders coupled with an unconditional monetary incentive on MCV1 timely coverage in this study—an approximately 9%–11% absolute increase—was less than the 21% absolute increase associated with SMS reminders coupled with conditional incentives in the M-SIMU study. Second, the finding that the effect of SMS reminders coupled with an unconditional incentive was similar to that of SMS reminders alone was in contrast to findings from the M-SIMU study where the effect of SMS reminders coupled with conditional incentives surpassed that of SMS reminders alone.^17^

There is mixed evidence on whether the impact of conditional incentives on health outcomes varies from that of unconditional incentives. Systematic reviews have found positive effects of both conditional and unconditional financial incentives for outcomes such as HIV prevention and treatment and healthcare seeking.^38–40^ Some studies have found that unconditional incentives improve return or response rates for health-related surveys whereas conditional incentives do not, though not all incentives were financial.^41–43^ In terms of vaccination outcomes, evaluations of large conditional and unconditional cash transfer programmes in Central and South America found that transfers significantly increased uptake for some childhood vaccines but not for others, with neither type of cash transfer programme having consistent effects.^44–47^ Three studies of de facto conditional (incentives were delivered at immunisation visits) non-cash incentives in Pakistan and India found that the incentives significantly increased DTP3 or overall vaccination uptake.^22 35 37^ In the one cluster randomised controlled trial that simultaneously assessed the impact of both conditional and unconditional cash transfers on childhood health in Zimbabwe, neither intervention significantly improved vaccination uptake.^48^ Contrary to the suggestion from this study that conditional incentives had a greater effect compared with unconditional incentives, conditional incentives have been theorised to negatively impact intrinsic motivation in the behavioural economics literature.^49^ Further research is needed to evaluate whether, in a setting where intrinsic motivation to practice the target behaviour is fairly high—in this setting coverage for third-dose DTP-containing vaccine was 96% (table 1)—conditional incentives may in the short run improve outcomes through increasing extrinsic motivation.

At the same time, methodological factors (and not conditionality of the incentive) may explain differences in the effects in this study compared with those observed previously in the M-SIMU study. Whereas SMS reminders plus conditional incentives were associated with a statistically significant 6% absolute increase in MCV1 coverage by age 12 months in the M-SIMU study, the 7% absolute increase in the SMS + 150 KES arm of this study was not statistically significant. However, this study was underpowered to detect the observed increase, as we assumed an absolute increase in MCV1 timely coverage of ≥15% in sample size calculations. To be sufficiently powered to detect the approximately 7% absolute increase in MCV1 coverage by age 12 months observed, a sample size of approximately 593 individuals in each study arm would have been required. Second, the incentive amount and nature of the incentive differed in this study compared with the M-SIMU study. The incentive amount in this study was KES 150 (~US$1.87 in August 2015; indexed to 2015 KES) compared with KES 200 (~US$2.35 in August 2015; indexed to 2015 KES) in the M-SIMU study. Furthermore, in this study, caregivers received a maximum monetary incentive of KES 150 compared with as much as KES 800 in the M-SIMU study as that study incentivised the pentavalent vaccine primary series in addition to MCV1.^17^ The higher incentive amount in the M-SIMU study may have been more motivating to caregivers given that the value of incentives is thought to be positively correlated to their effect.^49^ Furthermore, formative research for the M-SIMU study found that 93% of caregivers felt that KES 200 would motivate prioritising attending a vaccination visit compared with 83% for KES 150 (D. Gibson personal communication). Finally, the repeated vaccination prompts during the M-SIMU study (SMS reminders for the pentavalent series and MCV1) may have induced greater vaccine seeking among those caregivers as more frequent reminders may be associated with greater impact.^50^

Beyond research design, a nationwide nurses’ strike that began on 5 June 2017^51^ and was ongoing at the time of study completion, represented an unexpected supply-side barrier to vaccination which may have affected the study findings. Approximately three-quarters and one-third of the analytic sample reached 10 months of age and 12 months of age, respectively, before the strike began. We observed that the 78% MCV1 coverage estimate in the Control arm was lower than the expected 83%–84% coverage based on previous estimates of coverage in the study area^17 31^ and in Siaya County.^52^ Exclusion of infants that had received a dose of MCV prior to reaching age 9 months would not explain the discrepancy; had they been enrolled and followed up (a conservative assumption), Control MCV1 coverage would have been ~80%. Although MCV1 coverage was not significantly higher in intervention infants who reached age 12 months before the strike compared with control infants who also reached age 12 months before the strike (online supplemental table S6), in theory, the nurses’ strike may have reduced the potential impact of interventions if intervention arm caregivers seeking MCV1 in public health facilities were unable to get their infants vaccinated as a result of the strike. Of note, estimates of MCV1 timely coverage and coverage by age 12 months in the intervention arms in the period before the nurses’ strike were comparable (online supplemental table S6).

This study has several limitations. First, the analytic sample comprised 85% of enrolled participants. Excluded infants were more likely to be firstborn children and to have mothers aged 25 years or less. Similar patterns were observed previously and are attributed to cultural practices around pregnancy and birth whereby mothers return to their rural home to receive support caring for newly-born infants.^17^ Firstborn infants and children of younger mothers are thought to be less at risk of being unvaccinated or receiving vaccination with delay than later-born infants and children of older mothers, respectively.^31 53–56^ Thus, the effect estimates may have been biased towards the null. Another limitation of this study is that only 85% of SMS reminders and 61% of mMoney incentives were sent out as intended which may have biased the effect toward the null. Indeed, the per-protocol analysis but not the ITT analysis found SMS reminders significantly improved MCV1 timeliness. Delivery of SMS reminders was hampered mainly by power outages; therefore, the findings may provide some insight into the real-world effectiveness of SMS reminders when delivered within a programmatic setting likely to experience similar challenges. mMoney incentives were delayed because they were sent out manually by study staff during weekdays but not on weekends when staff were not working. In post hoc analyses, delayed delivery of the incentive did not impact MCV1 timely coverage (online supplemental tables S7 and S8). A related limitation is that≥20% of caregivers may not have received the interventions, based on self-report. If true, the observed effects would be biased towards the null. We did not have objective mechanisms to verify intervention receipt and self-report was susceptible to recall bias as we asked caregivers whether they had received the interventions about 3 months after they had been sent. Besides recall bias, reported receipt of interventions may have been influenced by phone ownership, whereby caregivers who owned a phone may have been more likely to remember having received the intervention compared with those sharing the phone to which interventions were sent. Curiously, the proportion of SMS + 150 KES caregivers who reported receiving SMS reminders was higher than the proportion who reported receiving the incentive. This could perhaps be explained if SMS messages were more likely to be passed along than the incentive for SMS + 150 KES caregivers who shared a phone. An additional limitation of this study is that COMM-Is, who collected measles vaccination status at the follow-up visit, were responsible for assigning the study arm at enrolment and were therefore not blinded to study arm allocation. We think ascertainment bias is unlikely because follow-up occurred 4–6 months from enrolment and we structured the follow-up questionnaire to collect vaccination data prior to any questions that identified the study arm allocation. Repeat follow-up interviews by the study’s field supervisor support this; of the 5% that was reinterviewed, only one vaccination discrepancy was found.

## Conclusion

This study’s findings suggest that the impact of SMS reminders coupled with a small unconditional monetary incentive on MCV1 timeliness was comparable to that of SMS reminders alone, yet it would be more costly to implement. In addition, the impact of SMS reminders coupled with small unconditional incentives on reducing the proportion of measles-unvaccinated infants was unclear, though the findings suggest that any effect is likely to be modest and of similar magnitude to that of SMS reminders alone. Notably, the similarity in MCV1 uptake levels among infants in the intervention arms was observed even before the nurses’ strike and other supply-side constraints observed in the study are likely to be experienced under real-world conditions in LMICs. Additional studies in the absence of unexpected supply-side constraints, such as the nurses’ strike, are needed to inform the generalisability of the findings.

## References

[1] Patel MK, Dumolard L, Nedelec Y, et al Progress Toward Regional Measles Elimination - Worldwide, 2000-2018. MMWR Morb Mortal Wkly Rep 2019;68:1105–11. 10.15585/mmwr.mm6848a1 31805033 PMC6897527

[2] World Health Organization (WHO) Global and regional immunization profile, 2020 Available: https://www.who.int/immunization/monitoring_surveillance/data/gs_gloprofile.pdf?ua=1 [Accessed 15 Oct 2020].

[3] World Health Organization (WHO) Global measles and rubella strategic plan: 2012-2020. Available: http://who.int/immunization/documents/control/ISBN_978_92_4_150339_6/en/ [Accessed February 7, 2017].

[4] World Health Organization (WHO) Global and regional immunization profile: African region, 2020 Available: https://www.who.int/immunization/monitoring_surveillance/data/gs_afrprofile.pdf?ua=1 [Accessed 15 Oct 2020].

[5] World Health Organization (WHO) Global and regional immunization profile: European region, 2020 Available: https://www.who.int/immunization/monitoring_surveillance/data/gs_eurprofile.pdf?ua=1 [Accessed 15 Oct 2020].

[6] World Health Organization (WHO) Global and regional immunization profile: south-east Asia region, 2020 Available: https://www.who.int/immunization/monitoring_surveillance/data/gs_searprofile.pdf?ua=1 [Accessed 15 Oct 2020].

[7] World Health Organization (WHO) Global and regional immunization profile: Western Pacific region, 2020 Available: https://www.who.int/immunization/monitoring_surveillance/data/gs_wprprofile.pdf [Accessed 15 Oct 2020].

[8] World Health Organization (WHO) Global health Observatory data Repository. Available: https://apps.who.int/gho/data/view.main.81100WB?lang=en [Accessed 17 Mar 2020].

[9] World Health Organization (WHO) State of inequality: childhood immunization. Available: http://who.int/gho/health_equity/report_2016_immunization/en/ [Accessed 23 Feb 2017].

[10] International Telecommunications Union (ITU) Key ICT indicators for developed and developing countries and the world (totals and penetration rates). Available: http://www.itu.int/en/ITU-D/Statistics/Pages/stat/default.aspx [Accessed 27 May 2020].

[11] Schlumberger M, Bamoko A, Yaméogo TM, et al Impact positif sur le Programme élargi de vaccinations de l’envoi de SMS de rappel à partir d’un registre informatisé, Bobo-Dioulasso (Burkina Faso). Bull Soc Pathol Exot 2015;108:349–54. 10.1007/s13149-015-0455-4 26498331

[12] Uddin MJ, Shamsuzzaman M, Horng L, et al Use of mobile phones for improving vaccination coverage among children living in rural hard-to-reach areas and urban streets of Bangladesh. Vaccine 2016;34:276–83. 10.1016/j.vaccine.2015.11.024 26647290 PMC4807732

[13] Bangure D, Chirundu D, Gombe N, et al Effectiveness of short message services reminder on childhood immunization programme in Kadoma, Zimbabwe - a randomized controlled trial, 2013. BMC Public Health 2015;15:137. 10.1186/s12889-015-1470-6 25885862 PMC4339642

[14] Kazi AM, Ali M, Zubair K, et al Effect of mobile phone text message reminders on routine immunization uptake in Pakistan: randomized controlled trial. JMIR Public Health Surveill 2018;4:e20. 10.2196/publichealth.7026 29514773 PMC5863012

[15] Eze GU, Adeleye OO Enhancing routine immunization performance using innovative technology in an urban area of Nigeria. West Afr J Med 2015;34:3–10. 26902809

[16] Haji A, Lowther S, Ngan’ga Z Reducing routine vaccination dropout rates: evaluating two interventions in three Kenyan districts, 2014. BMC Public Health 2016;16:152. 10.1186/s12889-016-2823-5 26880141 PMC4754928

[17] Gibson DG, Ochieng B, Kagucia EW, et al Mobile phone-delivered reminders and incentives to improve childhood immunisation coverage and timeliness in Kenya (M-SIMU): a cluster randomised controlled trial. Lancet Glob Health 2017;5:e428–38. 10.1016/S2214-109X(17)30072-4 28288747 PMC5348605

[18] Nguyen NT, Vu HM, Dao SD, et al Digital immunization registry: evidence for the impact of mHealth on enhancing the immunization system and improving immunization coverage for children under one year old in Vietnam. Mhealth 2017;3:26. 10.21037/mhealth.2017.06.03 28828373 PMC5547172

[19] Ekhaguere OA, Oluwafemi RO, Badejoko B, et al Automated phone call and text reminders for childhood immunisations (PRIMM): a randomised controlled trial in Nigeria. BMJ Glob Health 2019;4:e001232. 10.1136/bmjgh-2018-001232 PMC6509606 31139442

[20] Chen L, Du X, Zhang L, et al Effectiveness of a smartphone APP on improving immunization of children in rural Sichuan Province, China: a cluster randomized controlled trial. BMC Public Health 2016;16:909. 10.1186/s12889-016-3549-0 27581655 PMC5006404

[21] Kawakatsu Y, Oyeniyi Adesina A, Kadoi N, et al Cost-Effectiveness of SMS appointment reminders in increasing vaccination uptake in Lagos, Nigeria: a multi-centered randomized controlled trial. Vaccine 2020;38:6600–8. 10.1016/j.vaccine.2020.07.075 32788139

[22] Seth R, Akinboyo I, Chhabra A, et al Mobile phone incentives for childhood immunizations in rural India. Pediatrics 2018;141:1–3 http://pediatrics.aappublications.org/content/early/2018/03/12/peds.2017-3455.abstract 10.1542/peds.2017-3455 PMC5869335 29540571

[23] Kenya National Bureau of Statistics The 2009 Kenya population and housing census, 2010 Available: https://s3-eu-west-1.amazonaws.com/s3.sourceafrica.net/documents/21195/Census-2009.pdf [Accessed 24 Dec 2019].

[24] Kenya National Bureau of Statistics Distribution of population by sex and sub-county, 2020 Available: https://www.knbs.or.ke/?wpdmpro=2019-kenya-population-and-housing-census-volume-i-population-by-county-and-sub-county [Accessed 17 Oct 2020].

[25] Odhiambo FO, Laserson KF, Sewe M, et al Profile: the KEMRI/CDC Health and Demographic Surveillance System--Western Kenya. Int J Epidemiol 2012;41:977–87. 10.1093/ije/dys108 22933646 PMC12083774

[26] Schulz KF, Altman DG, Moher D Consort 2010 statement: updated guidelines for reporting parallel group randomised trials. J Clin Epidemiol 2010;63:63–840. 10.1016/j.jclinepi.2010.02.005 20346629

[27] Gibson DG, Kagucia EW, Were J, et al Text message reminders and unconditional monetary incentives to improve measles vaccination in Western Kenya: study protocol for the mobile and scalable innovations for measles immunization randomized controlled trial. JMIR Res Protoc 2019;8:e13221. 10.2196/13221 31290405 PMC6647752

[28] RapidSMS About RapidSMS. Available: https://www.rapidsms.org/about/ [Accessed 10 Jan 2018].

[29] Government of Kenya Ministry of Health National policy guidelines on immunization, 2013 Available: http://e-cavi.com/wp-content/uploads/2014/11/KENYA-NATIONAL-POLICY-ON-IMMUNIZATION-2013.pdf [Accessed 6 May 2018].

[30] Gibson DG, Kagucia EW, Ochieng B, et al The mobile solutions for immunization (M-SIMU) trial: a protocol for a cluster randomized controlled trial that assesses the impact of mobile phone delivered reminders and travel subsidies to improve childhood immunization coverage rates and timeliness in Western Kenya. JMIR Res Protoc 2016;5:e72. 10.2196/resprot.5030 27189422 PMC4887657

[31] Gibson DG, Ochieng B, Kagucia EW, et al Individual level determinants for not receiving immunization, receiving immunization with delay, and being severely underimmunized among rural Western Kenyan children. Vaccine 2015;33:6778–85. 10.1016/j.vaccine.2015.10.021 26482146

[32] Moss WJ, Fisher C, Scott S, et al Hiv type 1 infection is a risk factor for mortality in hospitalized Zambian children with measles. Clin Infect Dis 2008;46:523–7. 10.1086/526525 18194095

[33] Domek GJ, Contreras-Roldan IL, O'Leary ST, et al Sms text message reminders to improve infant vaccination coverage in Guatemala: a pilot randomized controlled trial. Vaccine 2016;34:2437–43. 10.1016/j.vaccine.2016.03.065 27026145 PMC4859823

[34] Garcia-Dia MJ, Fitzpatrick JJ, Madigan EA, et al Using text reminder to improve childhood immunization adherence in the Philippines. Comput Inform Nurs 2017;35:212–8. 10.1097/CIN.0000000000000307 27828815

[35] Banerjee AV, Duflo E, Glennerster R, et al Improving immunisation coverage in rural India: clustered randomised controlled evaluation of immunisation campaigns with and without incentives. BMJ 2010;340:c2220 http://www.bmj.com/content/340/bmj.c2220.abstract 10.1136/bmj.c2220 20478960 PMC2871989

[36] Briere EC, Ryman TK, Cartwright E, et al Impact of integration of hygiene kit distribution with routine immunizations on infant vaccine coverage and water treatment and handwashing practices of Kenyan mothers. J Infect Dis 2012;205:S56–64. 10.1093/infdis/jir779 22315387

[37] Chandir S, Khan AJ, Hussain H, et al Effect of food coupon incentives on timely completion of DTP immunization series in children from a low-income area in Karachi, Pakistan: a longitudinal intervention study. Vaccine 2010;28:3473–8. 10.1016/j.vaccine.2010.02.061 20199756

[38] Gaarder MM, Glassman A, Todd JE Conditional cash transfers and health: unpacking the causal chain. J Dev Effect 2010;2:6–50. 10.1080/19439341003646188

[39] Bassett IV, Wilson D, Taaffe J, et al Financial incentives to improve progression through the HIV treatment cascade. Curr Opin HIV AIDS 2015;10:451–63. 10.1097/COH.0000000000000196 26371461 PMC4699403

[40] Bassani DG, Arora P, Wazny K, et al Financial incentives and coverage of child health interventions: a systematic review and meta-analysis. BMC Public Health 2013;13:S30. 10.1186/1471-2458-13-S3-S30 24564520 PMC3847540

[41] Church AH Estimating the effect of incentives on mail survey response rates: a meta-analysis. Public Opin Q 1993;57:62–79. 10.1086/269355

[42] Young B, Bedford L, das Nair R, et al Unconditional and conditional monetary incentives to increase response to Mailed questionnaires: a randomized controlled study within a trial (SWAT). J Eval Clin Pract 2020;26:893–902. 10.1111/jep.13230 31328399

[43] Young JM, O'Halloran A, McAulay C, et al Unconditional and conditional incentives differentially improved general practitioners' participation in an online survey: randomized controlled trial. J Clin Epidemiol 2015;68:693–7. 10.1016/j.jclinepi.2014.09.013 25450450

[44] Morris SS, Flores R, Olinto P, et al Monetary incentives in primary health care and effects on use and coverage of preventive health care interventions in rural Honduras: cluster randomised trial. Lancet 2004;364:2030–7. 10.1016/S0140-6736(04)17515-6 15582060

[45] Attanasio O, Gomez L, Heredia P The short-term impact of a conditional cash subsidy on child health and nutrition in Colombia, 2005 Available: https://www.ifs.org.uk/edepo/rs_fam03.pdf [Accessed 3 Mar 2017].

[46] Barham T, Maluccio JA Eradicating diseases: the effect of conditional cash transfers on vaccination coverage in rural Nicaragua. J Health Econ 2009;28:611–21. 10.1016/j.jhealeco.2008.12.010 19233495

[47] Barham T The impact of the Mexican conditional cash transfer program on immunization rates, 2005 Available: http://www.colorado.edu/ibs/hs/barham/wp/CCTimmunWBfin.pdf [Accessed 19 Sep 2017].

[48] Robertson L, Mushati P, Eaton JW, et al Effects of unconditional and conditional cash transfers on child health and development in Zimbabwe: a cluster-randomised trial. Lancet 2013;381:1283–92. 10.1016/S0140-6736(12)62168-0 23453283 PMC3627205

[49] Gneezy U, Rustichini A Pay Enough or Don't Pay at All*. Q J Econ 2000;115:791–810 http://www.jstor.org/stable/2586896 10.1162/003355300554917

[50] Fry JP, Neff RA Periodic prompts and reminders in health promotion and health behavior interventions: systematic review. J Med Internet Res 2009;11:e16. 10.2196/jmir.1138 19632970 PMC2762806

[51] Daily Nation Nurses’ strike kicks off, premature babies suffer in Samburu. Available: https://www.nation.co.ke/news/Nurses-strike-kicks-off-in-Kenya/1056-3956290-13e90dd/index.html [Accessed 14 Jun 2017].

[52] Kenya National Bureau of Statistics, Ministry of Health/Kenya, National AIDS Control Council/Kenya, Kenya Medical Research Institute, National Council for Population and Development/Kenya and II Kenya demographic and health survey 2014, 2015 Available: http://dhsprogram.com/pubs/pdf/FR308/FR308.pdf [Accessed 18 Oct 2016].

[53] Griffin MR, Daugherty J, Reed GW, et al Immunization coverage among infants enrolled in the Tennessee Medicaid program. Arch Pediatr Adolesc Med 1995;149:559–64. 10.1001/archpedi.1995.02170180089017 7735413

[54] Matsumura T, Nakayama T, Okamoto S, et al Measles vaccine coverage and factors related to uncompleted vaccination among 18-month-old and 36-month-old children in Kyoto, Japan. BMC Public Health 2005;5:59. 10.1186/1471-2458-5-59 15935101 PMC1177963

[55] Schaffer SJ, Szilagyi PG Immunization status and birth order. Arch Pediatr Adolesc Med 1995;149:792–7. 10.1001/archpedi.1995.02170200082013 7795771

[56] Dayan GH, Shaw KM, Baughman AL, et al Assessment of delay in age-appropriate vaccination using survival analysis. Am J Epidemiol 2006;163:561–70. 10.1093/aje/kwj074 16421238

