# Supplementary material for: Impact of mobile phone delivered reminders and unconditional incentives on measles-containing vaccine timeliness and coverage: a randomised controlled trial in western Kenya
Source: BMJ Glob Health. 2021 Jan 28;6(1):e003357. doi: 10.1136/bmjgh-2020-003357 (PMC7845730; doi:10.1136/bmjgh-2020-003357)
Supplement: Supplementary data [file bmjgh-2020-003357supp001.pdf]

Supplement

Impact of mobile phone delivered reminders and unconditional incentives on measles-containing vaccine timeliness and coverage in western Kenya

Authors:
E. Wangeci Kagucia, PhD[1]; Benard Ochieng, MSc[2]; Joyce Were, BSc[2]; Kyla Hayford, PhD[1]; David Obor, MSc[2]; Katherine L O'Brien, MD[1]; Dustin G Gibson, PhD[1]

[1]Johns Hopkins Bloomberg School of Public Health, Department of International Health, Baltimore, MD, United States

[2]Kenya Medical Research Institute/Centers for Disease Control and Prevention Public Health and Research Collaboration, Kisumu, Kenya

Corresponding Author:
Eunice Kagucia, PhD
Johns Hopkins Bloomberg School of Public Health
615 North Wolfe Street
Baltimore, MD 21205
Phone: +1-617-281-4853
Email: ekagucia@jhu.edu

Kagucia EW, *et al. BMJ Global Health* 2021; 6:e003357. doi: 10.1136/bmjgh-2020-003357

Box S1: SMS reminder content[1]

Message Type	Message Timing	Arm 1: Control	Arm 2 : SMS Only	Arm 3 : SMS + 150 KES
**Enrollment message**	Enrollment	Thank you for enrolling Baby <BABY'S FIRST NAME> in M-SIMI study. The greatest wealth is health. This study is sponsored by KEMRI	Thank you for enrolling your child in the KEMRI M-SIMI study. You will get SMS reminders for Baby <BABY'S FIRST NAME>'s measles vaccination. The greatest wealth is health.	Thank you for enrolling your child in the KEMRI M-SIMI study. You will get SMS reminders for Baby <BABY'S FIRST NAME>'s measles vaccination. The greatest wealth is health.
**3 day reminder message**	Date of Birth + 9 months – 3 days	No message	Tell Mama <BABY'S FIRST NAME> that Measles vaccine is due this week. Most Gem babies get vaccinated, be one of them!	Tell Mama <BABY'S FIRST NAME> that Measles vaccine is due this week. We are sending 150ksh to assist with travel. Most Gem babies get vaccinated, be one of them!
**1 day reminder message**	Date of Birth + 9 months – 1 day	No message	Tell Mama <Baby Fname> that Measles vaccine is due this week. Go to the clinic if you haven't already. Vaccines save Kenyan babies lives.	Tell Mama <Baby Fname> that Measles vaccine is due this week. Go to the clinic if you haven't already. Vaccines save Kenyan babies lives.

Random allocation procedure
A list of 537 "Allocation IDs", i.e., one unique ID for each study arm allocation, were generated. The study group associated with each Allocation ID followed the random sequence generated. Allocation IDs were placed into five groups, one for each of the five study-employed Community Interviewers (CIs) involved in screening and enrollment as below:

Group 1	Group 2	Group 3	Group 4	Group 5
Allocation ID 1A – 108A	Allocation ID 1B – 108B	Allocation ID 1C – 107C	Allocation ID 1D – 107D	Allocation ID 1E – 107E

The Allocation ID sequence (random allocation to study group) was maintained within each batch of Allocation IDs issued to CIs as below:

Allocation ID	Study group in random sequence (example)
1A	SMS+150KES
2A	SMS+150KES
3A	Control
4A	SMS only
5A	Control
6A	Control

The Allocation ID and study arm were printed on a card and placed into an opaque envelope which was labelled with only the Allocation ID and sealed as below:

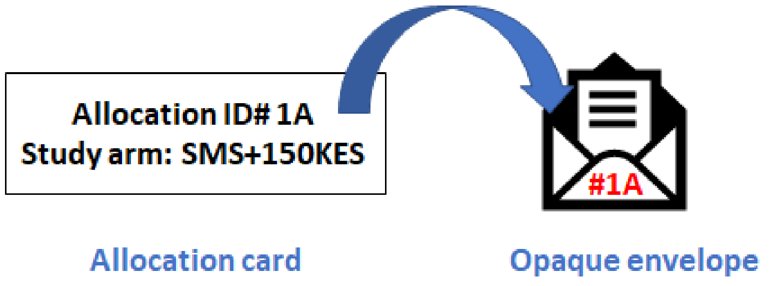

During the enrollment period, the site Principal Investigator (PI) provided CIs, who did not have access to the study group assignments, with a pre-defined number of allocation envelopes on a weekly basis as follows (example):

Kagucia EW, *et al. BMJ Global Health* 2021; 6:e003357. doi: 10.1136/bmjgh-2020-003357

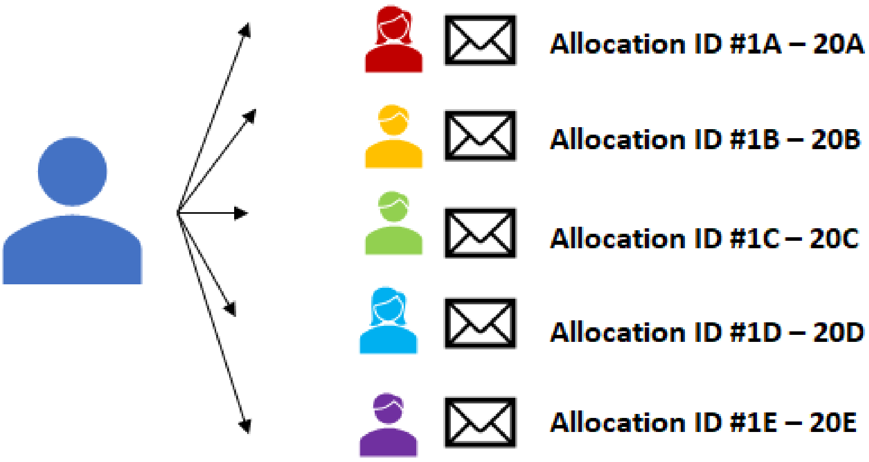

After determining that an infant was eligible for the study, the CI opened the allocation envelope to reveal the study arm. CIs opened each allocation envelope sequentially e.g., the allocation envelope labelled with Allocation ID #2A was opened after the allocation envelope labelled with Allocation ID #1A.

If a caregiver refused participation after the allocation envelope had been unsealed, the CI returned the unsealed allocation envelope to the PI. The Allocation ID of the unsealed envelope was then switched with that of a yet unused, sealed, randomly picked allocation envelope such that the study arm associated with the Allocation ID of the previously unsealed envelope had a chance of being different from the original study arm. Switching was performed to ensure that CIs continued to be blinded to the allocation sequence. The modified Allocation IDs and envelopes were then returned into circulation, as below:

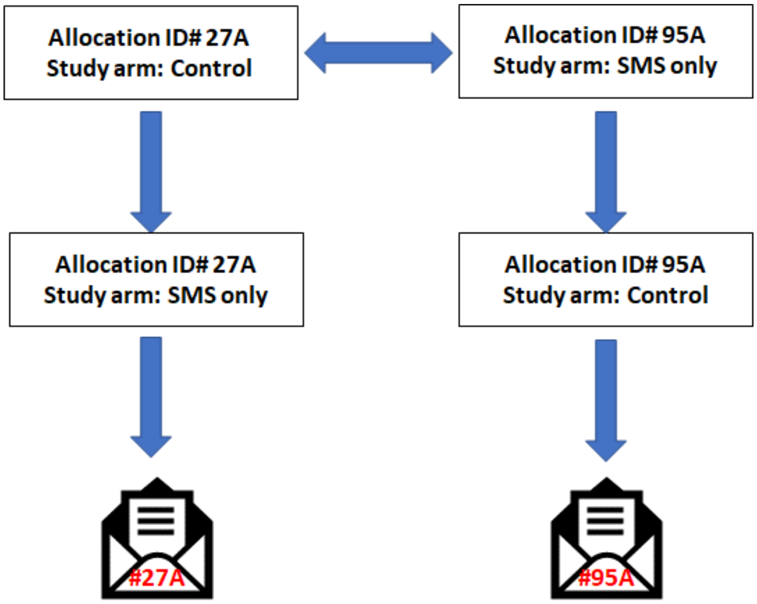

Table S1. Comparison of the distribution of baseline characteristics among infants in the analytic sample compared to excluded infants

	**Analytic sample (N= 455)**	**Verbal report (N= 24)**	**Lost to follow-up (N= 54)**	**Twins (N= 4)**	**All excluded (N= 82)**	**p-value***
	**No. (%)**	**No. (%)**	**No. (%)**		**No. (%)**	
**Mobile phone access**						
Shares	143 (31.4)	7 (29.2)	19 (35.2)	0	26 (31.7)	0.960
Owns	312 (68.6)	17 (70.8)	35 (64.8)	4 (100)	56 (68.3)	
**Infant's sex**						
Female	216 (47.5)	9 (37.5)	31 (57.4)	3 (75.0)	43 (52.4)	0.407
Male	239 (52.5)	15 (62.5)	23 (42.6)	1 (25.0)	39 (47.6)	
**Infant's age at enrollment**						
6m	292 (64.2)	15 (62.5)	32 (59.3)	0	47 (57.3)	0.206
7m	156 (34.3)	9 (37.5)	22 (40.7)	4 (100)	35 (42.7)	
8m	7 (1.5)	0	0	0	0	
**Penta3 before enrollment (MCH card record only)**						
Not vaccinated	18 (4.0)	3 (13.0)	3 (5.8)	0	6 (7.6)	0.150
Vaccinated	437 (96.0)	20 (87.0)	49 (94.2)	4 (100)	73 (92.4)	
**Penta3 before enrollment (MCH card or verbal record)**						
Not vaccinated	18 (4.0)	3 (12.5)	3 (5.6)	0	6 (7.3)	0.175
Vaccinated	437 (96.0)	21 (87.5)	51 (94.4)	4 (100)	76 (92.7)	
**Time to health facility**						
≤30 minutes	292 (64.2)	12 (50.0)	36 (66.7)	2 (50.0)	50 (61.0)	0.579
>30 minutes	163 (35.8)	12 (50.0)	18 (33.3)	2 (50.0)	32 (39.0)	
**Maternal education**						
≤7 years	156 (34.3)	10 (41.7)	15 (27.8)	2 (50.0)	27 (32.9)	0.811
>7 years	299 (65.7)	14 (58.3)	39 (72.2)	2 (50.0)	55 (67.1)	
**Birth order**						
Firstborn	86 (18.9)	7 (29.2)	27 (50.0)	0	34 (41.5)	**<0.001**
Later-born	369 (81.1)	17 (70.8)	27 (50.0)	4 (100)	48 (58.5)	
**Location of last delivery**						
At home	83 (18.3)	3 (12.5)	10 (18.5)	0	13 (15.9)	0.598
Health facility	371 (81.7)	21 (87.5)	44 (81.5)	4 (100)	69 (84.1)	
**Maternal age**						
≤25 years	228 (50.1)	12 (50.0)	42 (77.8)	2 (50.0)	56 (68.3)	**0.002**
>25 years	227 (49.9)	12 (50.0)	12 (22.2)	2 (50.0)	26 (31.7)	
**Number of ANC visits for enrolled infant**						
≤4 visits	309 (68.2)	17 (73.9)	43 (79.6)	4 (100)	64 (79.0)	0.051
>4 visits	144 (31.8)	6 (26.1)	11 (20.4)	0	17 (21.0)	
**Socioeconomic quintile**						

Kagucia EW, *et al. BMJ Global Health* 2021; 6:e003357. doi: 10.1136/bmjgh-2020-003357

	**Analytic sample (N= 455)**	**Verbal report (N= 24)**	**Lost to follow-up (N= 54)**	**Twins (N= 4)**	**All excluded (N= 82)**	**p-value***
	**No. (%)**	**No. (%)**	**No. (%)**		**No. (%)**	
Bottom 40%	179 (39.3)	9 (37.5)	27 (50.0)	0	36 (43.9)	0.438
Upper 60%	276 (60.7)	15 (62.5)	27 (50.0)	4 (100)	46 (56.1)	

*p-value for comparison of analytic sample to all excluded

Table S2. Univariate risk factor analysis for not receiving MCV1 by age 10 months among Control children

	MCV1 status			
	**Not vaccinated (N= 51) n (%)**	**Vaccinated (N= 109) n (%)**	**cRR (95% CI)**	**p-value**
**Owns phone**				
No	21 (42.9)	28 (57.1)	Ref	
Yes	30 (27.0)	81 (73.0)	1.28 (0.98, 1.67)	**0.073**
**Infant's sex**				
Female	25 (32.5)	52 (67.5)	Ref	
Male	26 (31.3)	57 (68.7)	1.02 (0.82, 1.26)	0.877
**Infant's age at enrollment**				
6m	38 (36.5)	66 (63.5)	Ref	
7-8m	13 (23.2)	43 (76.8)	1.21 (0.99, 1.49)	**0.068**
**Penta3 before enrollment (MCH card record only)**				
No	4 (80.0)	1 (20.0)	Ref	
Yes	47 (30.3)	108 (69.7)	3.48 (0.60, 20.17)	0.164
**Time to health facility**				
≤30 minutes	36 (32.7)	74 (67.3)	Ref	
>30 minutes	15 (30.0)	35 (70.0)	1.04 (0.83, 1.30)	0.727
**Maternal education**				
≤7 years	21 (39.6)	32 (60.4)	Ref	
>7 years	30 (28.0)	77 (72.0)	1.19 (0.93, 1.53)	0.166
**Birth order**				
Firstborn	7 (21.2)	26 (78.8)	Ref	
Later-born	44 (34.6)	83 (65.4)	0.83 (0.67, 1.03)	**0.092**
**Location of last delivery**				
At home	11 (36.7)	19 (63.3)	Ref	
Health facility	40 (31.0)	89 (69.0)	1.09 (0.81, 1.46)	0.571
**Maternal age**				
≤25 years	23 (28.7)	57 (71.3)	Ref	
>25 years	28 (35.0)	52 (65.0)	0.91 (0.74, 1.13)	0.398
**Number of ANC visits for enrolled infant**				

	MCV1 status			
	**Not vaccinated (N= 51) n (%)**	**Vaccinated (N= 109) n (%)**	**cRR (95% CI)**	**p-value**
≤4 visits	40 (35.1)	74 (64.9)	Ref	
>4 visits	11 (23.9)	35 (76.1)	1.17 (0.95, 1.45)	0.140
**Socioeconomic quintile**				
Bottom 40%	22 (30.6)	50 (69.4)	Ref	
Upper 60%	29 (33.0)	59 (67.0)	0.96 (0.78, 1.19)	0.745

Table S3. Number and timing of SMS reminders by intervention study arm and overall

SMS delivery	SMS N=146 n (%)	SMS+150KES N=149 n (%)	Total N=295 n (%)
Per-protocol*	126 (86.3)	126 (84.6)	252 (85.4)
Not per-protocol	20 (13.7)	23 (15.4)	43 (14.6)
3 reminders sent	0 (0)	2 (8.7)	2 (4.6)
Reminder before day of appointment not sent	7 (35.0)	7 (30.4)	14 (32.5)
Reminder three days before appointment not sent	4 (20.0)	7 (30.4)	11 (25.6)
Second reminder sent on day of appointment	7 (35.0)	6 (26.1)	13 (30.2)
Reminders sent 2 days before and on day of appointment	1 (5.0)	0 (0)	1 (2.3)
Reminders sent after appointment date	1 (5.0)	1 (4.3)	2 (4.8)

*Per-protocol SMS delivery = 2 reminders sent, one three days before and the other one day before scheduled vaccination date

Kagucia EW, *et al. BMJ Global Health* 2021; 6:e003357. doi: 10.1136/bmjgh-2020-003357

Table S4. Distribution of timing of incentive payments relative to the scheduled vaccination date for SMS+150KES arm participants

When M-PESA sent*	Number of participants (%)
Day -3	91 (61.1)
Day -2	15 (10.1)
Day -1	21 (14.1)
Day 0	18 (12.1)
Day +1	1 (0.7)
Day +8	1 (0.7)
Day +10	1 (0.7)
Day +28	1 (0.7)
Total	149 (100)

*Reference point is scheduled vaccination date. For example, Day -3 is three days before scheduled vaccination date

Table S5. MCV1 uptake among infants of caregivers who cashed out the mMoney incentive

	**mMoney incentive cashed out**
**MCV1 by age 10 months**	
**Yes**	83 (81%)
**No**	20 (19%)
**MCV1 by age 12 months**	
**Yes**	90 (87%)
**No**	13 (13%)

Table S6. MCV1 uptake and intervention effect sizes before and after the beginning of the nurses' strike

**Before strike**						
**Study arm**	**MCV1 timely coverage**	**Adjusted RR**	**p-value**	**MCV1 coverage by age 12 months**	**Adjusted RR**	**p-value**
**Control (n= 160)**	71% (86/ 121)	Ref	--	87% (47/ 54)	Ref	--
**SMS (n= 146)**	82% (94/ 115)	1.13 (0.99, 1.30)	0.078	91% (39/ 43)	1.05 (0.92, 1.20)	0.462
**SMS+150 (n= 149)**	80% (86/ 107)	1.14 (0.99, 1.32)	0.064	88% (43/ 49)	1.00 (0.87, 1.16)	0.967
**After strike**						
**Study arm**	**MCV1 timely coverage**	**Adjusted RR**	**p-value**	**MCV1 coverage by age 12 months**	**Adjusted RR**	**p-value**
**Control (n= 160)**	59% (23/ 39)	Ref	--	74% (78/ 106)	Ref	--
**SMS (n= 146)**	65% (20/ 31)	1.10 (0.76, 1.60)	0.614	82% (84/ 103)	1.10 (0.95, 1.27)	0.207
**SMS+150 (n= 149)**	71% (30/ 42)	1.20 (0.86, 1.67)	0.278	83% (83/ 100)	1.14 (0.98, 1.31)	0.081

Table S7. Effect of per-protocol transmission of mMoney incentives on MCV1 timely coverage

mMoney transmission	N	Number vaccinated (%)	Crude RR	p-value
**Not per protocol**	58	46 (79%)	Ref	--
**Per protocol***	91	70 (77%)	0.97 (0.82, 1.15)	0.729

*Per protocol= Incentive sent three days before the scheduled MCV1 date

Table S8. Effect of modified per-protocol transmission of mMoney incentives on MCV1 timely coverage

mMoney transmission	N	Number vaccinated (%)	Crude RR	p-value
**Delayed**	22	17 (77%)	Ref	--
**Modified per protocol***	127	99 (78%)	1.01 (0.79, 1.29)	0.944

*Modified per protocol= Incentive sent before the scheduled MCV1 date

Kagucia EW, *et al. BMJ Global Health* 2021; 6:e003357. doi: 10.1136/bmjgh-2020-003357

Figure S1. Incidence of MCV1 vaccination with time of origin as age at enrollment. Events are censored at age 365 days

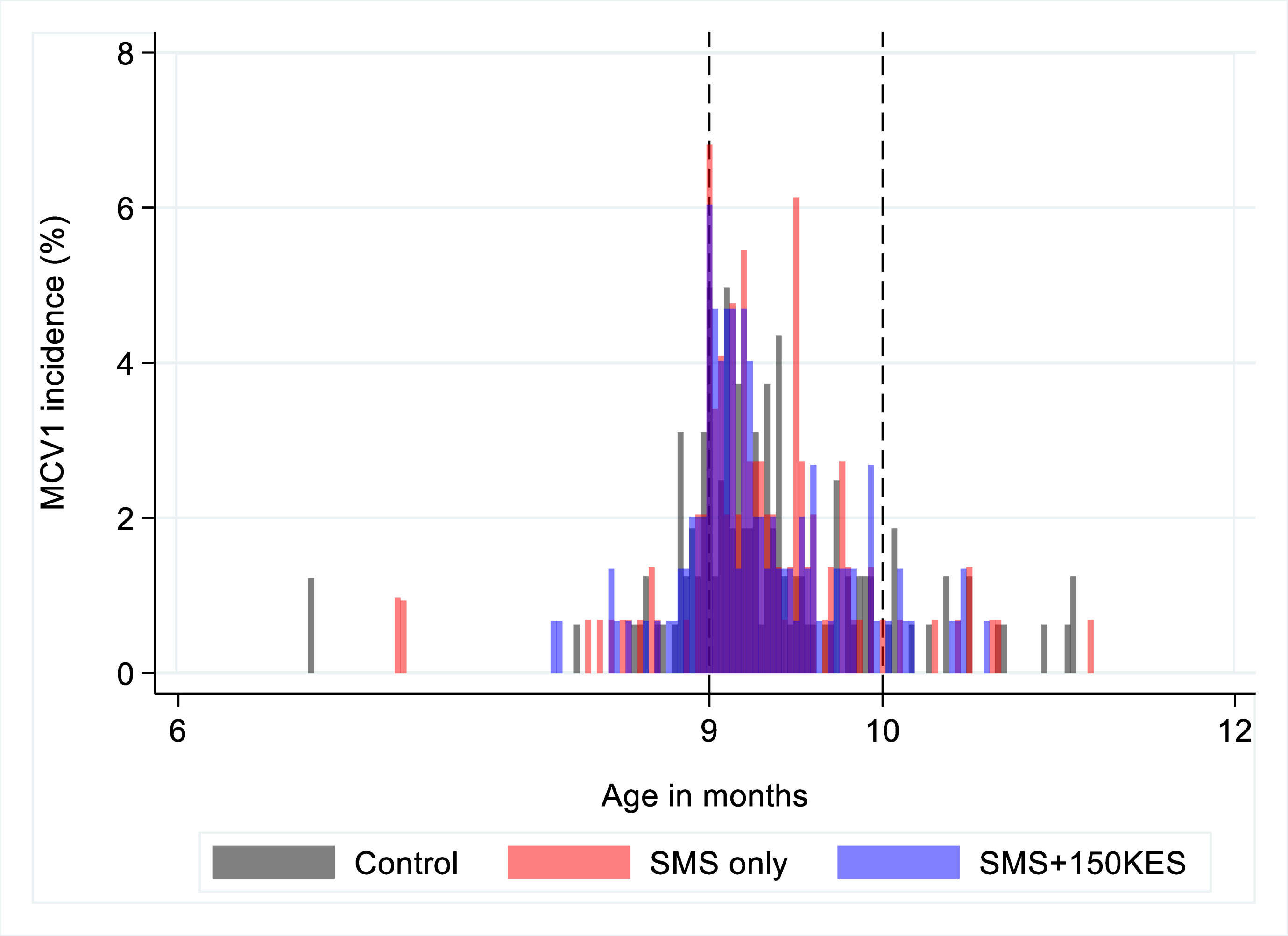

Kagucia EW, *et al. BMJ Global Health* 2021; 6:e003357. doi: 10.1136/bmjgh-2020-003357

Reference

1. Gibson DG, Kagucia EW, Were J, Obor D, Hayford K, Ochieng B. Text Message Reminders and Unconditional Monetary Incentives to Improve Measles Vaccination in Western Kenya: Study Protocol for the Mobile and Scalable Innovations for Measles Immunization Randomized Controlled Trial. *JMIR Res Protoc*. 2019;8(7):e13221. doi:10.2196/13221
